# The voltage-dependent K^+^ channels Kv1.3 and Kv1.5 in human cancer

**DOI:** 10.3389/fphys.2013.00283

**Published:** 2013-10-10

**Authors:** Núria Comes, Joanna Bielanska, Albert Vallejo-Gracia, Antonio Serrano-Albarrás, Laura Marruecos, Diana Gómez, Concepció Soler, Enric Condom, Santiago Ramón y Cajal, Javier Hernández-Losa, Joan C. Ferreres, Antonio Felipe

**Affiliations:** ^1^Molecular Physiology Laboratory, Departament de Bioquímica i Biologia Molecular, Institut de Biomedicina, Universitat de BarcelonaBarcelona, Spain; ^2^Departament de Patologia i Terapèutica Experimental, Hospital Universitari de Bellvitge-IDIBELL, L'Hospitalet de LlobregatBarcelona, Spain; ^3^Departament de Anatomía Patològica, Hospital Universitari Vall d'Hebron, Universitat Autònoma de BarcelonaBarcelona, Spain

**Keywords:** K^+^ channels, cancer, agressiveness, tumor markers, proliferation

## Abstract

Voltage-dependent K^+^ channels (Kv) are involved in a number of physiological processes, including immunomodulation, cell volume regulation, apoptosis as well as differentiation. Some Kv channels participate in the proliferation and migration of normal and tumor cells, contributing to metastasis. Altered expression of Kv1.3 and Kv1.5 channels has been found in several types of tumors and cancer cells. In general, while the expression of Kv1.3 apparently exhibits no clear pattern, Kv1.5 is induced in many of the analyzed metastatic tissues. Interestingly, evidence indicates that Kv1.5 channel shows inversed correlation with malignancy in some gliomas and non-Hodgkin's lymphomas. However, Kv1.3 and Kv1.5 are similarly remodeled in some cancers. For instance, expression of Kv1.3 and Kv1.5 correlates with a certain grade of tumorigenicity in muscle sarcomas. Differential remodeling of Kv1.3 and Kv1.5 expression in human cancers may indicate their role in tumor growth and their importance as potential tumor markers. However, despite of this increasing body of information, which considers Kv1.3 and Kv1.5 as emerging tumoral markers, further research must be performed to reach any conclusion. In this review, we summarize what it has been lately documented about Kv1.3 and Kv1.5 channels in human cancer.

## Voltage-dependent K^+^ channels Kv1.3 and Kv1.5

Potassium channels are one of the most diverse and ubiquitous families of membrane proteins and are encoded by more than 75 different genes (Caterall et al., 2002). Voltage-dependent K^+^ channels (Kv), a superfamily comprised of 12 subfamilies (Kv1-Kv12), play a key role in the maintenance of resting membrane potential and the control of action potentials (Hille, 2001). Kv channels also contribute to a wide variety of cellular processes including the maintenance of vascular smooth muscle tone (Yuan et al., 1998), cell growth (DeCoursey et al., 1984), the regulation of cell volume (Deutsch and Lee, 1988), adhesion (Itoh et al., 1995), mobility, epithelial transport (Kupper et al., 1995), homeostasis (Xu et al., 2003), insulin release (Xu et al., 2004), and apoptosis (Storey et al., 2003). Kv channels also control leukocyte membrane potential and play a role in immune system responses (Cahalan and Chandy, 2009). Accordingly, several studies have reported that Kv channels are involved in the activation, proliferation, differentiation, and migration of leukocytes (Cahalan and Chandy, 1997; Wulff et al., 2003; Panyi et al., 2004; Beeton et al., 2005; Felipe et al., 2006). Given their pivotal role in cell physiology, abnormalities in Kv functions can lead to several channelopathies (Ashcroft, 2000).

The voltage dependent K^+^ channels Kv1.3 and Kv1.5 are members of the *Shaker* (Kv1) family of K^+^ channels and are implicated in tissue differentiation and cell growth (Felipe et al., 2006). Although Kv1.3 was first cloned from brain tissue, its expression is widely distributed throughout the body (Swanson et al., 1990; Bielanska et al., 2009, 2010). This channel is highly expressed in lymphocytes and the olfactory bulb (Stuhmer et al., 1989), and several studies have reported that it is also expressed in the hippocampus (Veh et al., 1995), epithelia (Grunnet et al., 2003), adipose tissue (Xu et al., 2004), and both skeletal, and smooth muscle (Villalonga et al., 2008; Bielanska et al., 2012a,b).

Kv1.3 currents exhibit a characteristic cumulative inactivation and a marked C-type inactivation. The single channel conductance of Kv1.3 is 13 pS, and the voltage required for activation is −35 mV. In contrast, the Kv1.5 channel was first isolated from the human ventricle and is also expressed in the atria (Tamkun et al., 1991). Similar to the Kv1.3 channel, Kv1.5 is also ubiquitously expressed (Swanson et al., 1990; Bielanska et al., 2009, 2010). For example, Kv1.5 is expressed in the immune system, the kidney, skeletal and smooth muscle and, to a lesser extent, the brain (Coma et al., 2003; Vicente et al., 2003, 2006; Villalonga et al., 2008; Bielanska et al., 2012a,b). Kv1.5 currents contribute to the ultra-rapid activating K^+^ current in the heart known as I_kur_, which plays a role in the repolarization of an action potential (Lesage et al., 1992). The conductance of the Kv1.5 channel is 8 pS, and the voltage required for activation is ~24 mV. Unlike Kv1.3, Kv1.5 inactivation is slow and lacks cumulative inactivation. Such a different biophysical features may explain their distinct regulation in a number of cell types.

Kv1.3 and Kv1.5 are inhibited by 4-aminopyridine (4-AP) and tetraethylammonium (TEA), which are general K^+^ channel blockers (Grissmer et al., 1994). Psora-4 is another potent chemical inhibitor of both Kv1.3 and Kv1.5 and has a comparatively lesser effect on the rest of the Kv isoforms (Vennekamp et al., 2004). Highly specific toxins such as charybdotoxin and margatoxin (Leonard et al., 1992; Garcia-Calvo et al., 1993) as well as the anemone peptide ShK and their derivatives (Cahalan and Chandy, 1997) have proven to be highly effective for Kv1.3. On the other hand, Kv1.5 is highly insensitive to Kv1.3 blockers and has no known specific pharmacology. However, new chemicals such as S0100176 (from Sanofi-Aventis) (Decher et al., 2004) or diphenyl phosphine oxide-1 (DPO-1) have been discovered to potently inhibit Kv1.5 (Du et al., 2010).

Leukocytes express a diverse and unique repertoire of Kv proteins, however, Kv1.3 and Kv1.5 are considered the major Kv channels (Cahalan et al., 2001; Vicente et al., 2003, 2006; Wulff et al., 2004; Beeton et al., 2005; Cahalan and Chandy, 2009; Rangaraju et al., 2009; Sole et al., 2009; Felipe et al., 2010). In macrophages, dendritic cells and B lymphocytes, Kv currents are mainly mediated by Kv1.3, however, in contrast to T-lymphocytes, they also express Kv1.5 (Douglass et al., 1990; Vicente et al., 2003, 2006; Wulff et al., 2004; Mullen et al., 2006; Villalonga et al., 2007a,b; Zsiros et al., 2009; Villalonga et al., 2010a,b). We have previously shown that Kv1.5 subunits can coassemble with Kv1.3 subunits to generate functional heterotetrameric channels in macrophages. Interestingly, changes in the stoichiometry of the heterotetramers lead to the formation of new channels, which display different biophysical and pharmacological properties and influence the activation of specific cellular responses (Vicente et al., 2003, 2006, 2008; Villalonga et al., 2007a,b). The voltage for activation of Kv1.3 channel is more hyperpolarized than for Kv1.5. Thus, at physiological membrane potentials of most mammalian cells (from −30 to −60 mV), Kv1.3/Kv1.5 heteromeric channels with a high Kv1.3 ratio would be much more activated than those with low ratios of Kv1.3. The distinct voltage activation threshold of the two channels would explain why different subunit composition in Kv1.3/Kv1.5 complexes can lead to specific alteration of cellular excitability and determine different cell responses. Thus, the expression level of both subunits can influence the degree of cell proliferation, differentiation or activation. In this context, the Kv1.3/Kv1.5 ratio may be an accurate indicator of cell activation. For example, high levels of Kv1.5 would suggest a cell was maintaining an immunosuppressive state, whereas increased ratios of Kv1.3/Kv1.5 might indicate cell activation (Villalonga et al., 2007a,b; Felipe et al., 2010; Villalonga et al., 2010a,b). Leukocytes also express several regulatory subunits (Vicente et al., 2005; Sole et al., 2009) which may associate with Kv1.3/Kv1.5 complexes to enhance diversity and modulate a wide variety of physiological activities (McCormack et al., 1999). In fact, both channels Kv1.3 and Kv1.5, are able to assemble with Kvβ subunits to form functional Kv channels. Kvβ subunits alter current amplitude and gating, confer rapid inactivation, and promote Kv surface expression (Nakahira et al., 1996; Sewing et al., 1996; McCormack et al., 1999). In addition, heterologous expression of Kv1.3 and Kv1.5 with Kvβ subunits in *Xenopus* oocytes and mammalian cells, dramatically modifies the rate of inactivation (Sewing et al., 1996) and the K^+^ current density (McCormack et al., 1999), respectively.

Although most studies have been performed in adult tissues, Kv channels are differentially expressed throughout development. To date, several important differences in Kv expression during neonatal development have been reported (Roberds and Tamkun, 1991; Lesage et al., 1992; Felipe et al., 1994; Coma et al., 2002; Grande et al., 2003; Tsevi et al., 2005). We have recently studied the expression pattern of Kv1.3 and Kv1.5 in detail during the early stages of human development, and we have noted the following observations: (1) numerous tissues express Kv1.3 and Kv1.5 channels, (2) both channels are abundantly expressed in fetal liver (Bielanska et al., 2010), which serves as a hematopoietic tissue during early gestation, (3) adult hepatocytes did not express Kv1.3 (Vicente et al., 2003), (4) Kv1.5 is strongly expressed in fetal muscle and heart, whereas Kv1.3 abundance is low, (5) human fetal skeletal muscle expresses slightly more Kv1.3 than adult muscle fibers (Bielanska et al., 2010), and (6) the Kv1.5 channel is predominantly located in adult skeletal muscle and exhibits a cell cycle-dependent regulation pattern (Villalonga et al., 2008). We also examined brain tissue because it undergoes profound changes during the early fetal stages, such as cell proliferation, differentiation and migration. Kv1.3 localizes to the central and peripheral nervous systems, while Kv1.5 overlaps mostly with the central nervous system (Bielanska et al., 2010). In summary, we concluded that Kv1.3 and Kv1.5 channels followed a differential developmental expression profile, which eventually defines an adult phenotype and influences final physiological functions (Roberds and Tamkun, 1991; Lesage et al., 1992).

## The role of Kv1.3 and Kv1.5 in cell proliferation

Accumulating evidence suggests that many drugs and toxins that specifically block the activity of Kv channels decrease cell proliferation (Amigorena et al., 1990; Day et al., 1993; Wonderlin and Strobl, 1996; Chittajallu et al., 2002; Conti, 2004; Pardo, 2004; Kunzelmann, 2005; Felipe et al., 2006; Arcangeli et al., 2009; Wulff et al., 2009). For example, non-specific K^+^ channel blockers such as 4-AP, TEA and quinidine exert anti-proliferative effects in several different mammalian cell models (Mauro et al., 1997; Hoffman et al., 1998; Liu et al., 1998; Vaur et al., 1998; Wohlrab and Markwardt, 1999; Faehling et al., 2001; Wohlrab et al., 2002; Roderick et al., 2003).

Although the underlying mechanisms regarding how these channels promote proliferation is still a subject of debate (Roura-Ferrer et al., 2008; Villalonga et al., 2008), there are several events that may be controlled by Kv during cell growth, including membrane potential, Ca^2+^ signaling and cell volume (Wonderlin and Strobl, 1996; Conti, 2004; Pardo, 2004; Felipe et al., 2006). For example, during the early phases of cell cycle progression (G1/S), cells undergo a transient hyperpolarization which involves Kv channel activity (Wonderlin and Strobl, 1996). Interestingly, cancer cells are typically more depolarized in comparison with terminally differentiated cells (Pardo, 2004; O'Grady and Lee, 2005), although a transient hyperpolarization is required for the progression of the early G1 phase of the cell cycle (Wonderlin and Strobl, 1996). Thus, one would hypothesize that a blockage of K^+^ flux, which would lead to depolarization, should interfere with cell proliferation by inhibiting transient hyperpolarization.

Conversely, during lymphocyte proliferation, The combined action of Kv1.3 and KCa1 provides enough hyperpolarization to allow the Ca^2+^ influx required for proliferation. The resultant negative shift in membrane potential generates the required driving force for Ca^2+^ entry through Ca^2+^ channels (CRAC) from the extracellular space and its release from the inner stores (Arcangeli et al., 2009). Furthermore, cell growth is associated with cell volume increases throughout the G1 phase of the cell cycle (Lang et al., 2000). In fact, glioma cells exhibit their highest proliferation rates within a relatively narrow range of cell volumes, with decreased proliferation both over and under this optimal range. So, the rate of cell proliferation it is optimal within a cell volume window and appears to be controlled by low and high cell size checkpoints (Rouzaire-Dubois et al., 2004). Changes in membrane potential and cell volume are necessary for cell cycle progression, both of which require the action of K^+^ channels (Pardo, 2004).

The role of Kv channels in cell growth has been extensively studied in cells of the immune system. For instance, Kv1.3 is known to play an essential function in the activation of T lymphocytes (Panyi, 2005), which is dependent upon an increase in voltage-gated K^+^ conductance (McCormack et al., 1999). Moreover, a selective inhibition of Kv1.3 channels prevents cell activation and has been shown to exhibit immunosuppressive effects (Shah et al., 2003). Although previous studies have argued against a role of Kv1.3 in proliferation and activation of B-lymphocytes (Amigorena et al., 1990; Partiseti et al., 1993), it has been published that Kv1.3 protein levels increase in proliferating hippocampal microglia and control macrophage proliferation (Kotecha and Schlichter, 1999; Vicente et al., 2003; Villalonga et al., 2007a,b). Kv1.5 channels also play a crucial role in the activation and proliferation of oligodendrocytes, hippocampal microglia, macrophages and myoblasts (Attali et al., 1997; Kotecha and Schlichter, 1999; Villalonga et al., 2007a,b, 2008, 2010a,b). In macrophages, Kv1.3 depletion impairs cell growth and migration, both of which are characteristic features of cancer development (Villalonga et al., 2010a,b). Recently, we have determined that Kv1.5 is involved in the proliferation and migration of human B-cells (Vallejo-Gracia et al., 2013).

Several studies have demonstrated that Kv1.5 channels play a definitive role in muscle cell signaling. In this context, we have reported that regulation of Kv1.3 and Kv1.5 expression is cell cycle-dependent in L6E9 myoblasts. In fact, Kv1.5 expression changes throughout cell cycle progression with maximum expression occurring during the G1/S phase (Villalonga et al., 2008) and increased expression has also been noted during myogenesis (Vigdor-Alboim et al., 1999). Furthermore, our pharmacological evidence implies a role for Kv1.5 in the cell proliferation process (Villalonga et al., 2008). An alternative theory suggests that the role of the Kv1.3 channel in skeletal muscle could be connected to insulin sensitivity (Xu et al., 2004). However, Kv1.5 is thought to inhibit skeletal muscle cell proliferation through a mechanism involving the accumulation of cyclin-dependent kinase inhibitors (such as p21^cip−1^ and p27^kip1^) and a marked decrease in the expression of cyclins A and D1 (Villalonga et al., 2008).

It is well established that glial cells abundantly express Kv channels, including those that are part of the *Shaker* (Kv1) subfamily (Verkhratsky and Steinhauser, 2000), and different Kv channels are closely related to the cell cycle progression of human glia (Sontheimer, 1994). For instance, in rat oligodendrocyte precursor cells, the transition of quiescent cells into the G1 phase of the cell cycle is accompanied by increased levels of Kv1.3 and Kv1.5 proteins (Chittajallu et al., 2002). Moreover, the specific inhibition of Kv1.3 elicited a G1 arrest, while a reduction in Kv1.5 protein mediated by antisense oligonucleotide transfection had no effect on cell growth (Attali et al., 1997; Chittajallu et al., 2002). In contrast, Kv1.5 antisense treatment inhibited cell growth in astrocytes (MacFarlane and Sontheimer, 2000). Because blockage of Kv1.5 sufficiently decreased the proliferation of astrocytes but not oligodendrocytes, this channel may play different functional roles in different types of cells. In fact, these differential results, together with the involvement of Kv1.5 in cell growth (Attali et al., 1997; MacFarlane and Sontheimer, 2000; Soliven et al., 2003; Wang, 2004; Lan et al., 2005; Villalonga et al., 2008), argue against a singular role for these channels in cell proliferation. In addition, both Kv1.3 and Kv1.5 have been shown to be involved in promoting apoptosis. Psora-4, PAP-1 and clofazimine, three distinct membrane-permeable inhibitors of Kv1.3, induce cell death by directly targeting the mitochondrial channel in multiple human and mouse cancer cell lines (Leanza et al., 2012) and efficiently induce apoptosis of chronic lymphocytic leukemia cells (Leanza et al., 2013).

There is no clear understanding how K^+^ channels actually promote cell proliferation but possible mechanisms such as membrane voltage changes, cell volume regulation and the effect of mitogenic signals have been proposed (Wonderlin and Strobl, 1996). Exists a correlation between membrane potential and mitotic activity. Thus, terminally differentiated cells in G0 phase are very hyperpolarized whereas rapidly cycling tumor cells never entering G0 and are very depolarized (Binggeli and Weinstein, 1986). Mitogenic stimulation induces a short hyperpolarization at early G1, followed by depolarization. Although subsequent hyperpolarization during G1 has not been reported for all cell types, it is frequently observed and is believed to be essential for proliferation. Changes in membrane potential also alters the [Ca^2+^]_i_ concentration and promotes nutrient transport. Cell growth (increase in cell size) and proliferation (increase in number) are also closely related processes (Rouzaire-Dubois and Dubois, 1998). Thus, during progression through the cell cycle, the cell volume continuously changes. Particularly during G1/S transition and around the M phase large volume changes occur. It affects the concentration of enzymes that controls cell growth. Alteration in cell volume also regulate concentration of nutrients as well as cell-cycle effectors. Finally, cell cycle is controlled by distinct effectors such as oscillating cyclins and cyclin-dependent kinases. Inhibition of K^+^ currents causes membrane depolarization and accumulation of the cyclin-dependent kinase inhibitors p27 and p21 (Ghiani et al., 1999). Thus, cell cycle-relevant proteins may be directly regulated by membrane voltage. Current evidence points that voltage-sensitive K^+^ channels control cancer cell proliferation but the pathways involved are still unclear.

Same channels can participate in the stimulation of both cell proliferation and apoptosis. This paradox may depend on the temporal pattern of K^+^ channel activation. Thus, oscillating K^+^ channel activity typical of proliferating cells has completely different effects as sustained K^+^ channel activation typical of apoptotic cells. Activation of K^+^ channels during apoptosis is much more pronounced than during proliferation causing a drastic fall in the [K^+^]_*i*_ compared to during cell cycling (Cain et al., 2001; Bock et al., 2002). Since many of the growth-and mitosis-related enzymes require a minimal [K^+^]_i_, a loss of K^+^ reduce the proliferative activity (Hughes et al., 1997; Bortner and Cidlowski, 1999; Cain et al., 2001; Bock et al., 2002). Therefore, activation of both Cl^−^ and K^+^ channels must stay within a certain conductance to support proliferation, otherwise programmed cell death is triggered (Bock et al., 2002). Another important factor could be in Ca^2+^ signaling. Oscillatory Ca^2+^ rises were associated with proliferation and have not been observed during apoptosis. In contrast, a steady Ca^2+^ increase appears to be needed for apoptotic enzymes activity (Kunzelmann, 2005).

## Kv1.3 and Kv1.5 in solid cancers

In addition to their role in proliferation, migration and invasion (Conti, 2004; Pardo, 2004; Felipe et al., 2006; Villalonga et al., 2010a,b), potassium channels appear to contribute to the development of cancer (Kunzelmann, 2005). Kv channels are expressed in a number of tumors and cancer cell lines (Nilius and Wohlrab, 1992; Chin et al., 1997; Skryma et al., 1997; Laniado et al., 2001). Moreover, induced tumors in experimental models also exhibit high levels of several voltage-gated K^+^ channels, including Kv1.3 and Kv1.5 (Villalonga et al., 2007a,b).

Over the past decade, many studies have found that these channels are aberrantly expressed in different human tumor cells (Table 1), and the expression of both Kv1.3 and Kv1.5 channels is altered in a variety of human cancers including prostate cancer (Abdul and Hoosein, 2002a,b, 2006), colon cancer (Abdul and Hoosein, 2002a,b), breast cancer (Abdul et al., 2003; Brevet et al., 2009; Liu et al., 2010), lung cancer (Pancrazio et al., 1993; Wang et al., 2002), liver cancer (Zhou et al., 2003), smooth muscle cancers (Bielanska et al., 2012a), skeletal muscle cancers (Bielanska et al., 2012b), kidney cancer, bladder cancer, skin cancers (Bielanska et al., 2009) and gliomas (Preußat et al., 2003).

**Table 1:** **Expression of Kv1.3 and Kv1.5 in solid cancers and tumoral cells**.

**Tissues**	**Tumors and cell lines**	**Features**	**Kv1.3**	**Kv1.5**	**References**
Stomach	Stomach cancer epithelium cells	Positive in infiltrating inflammatory cells	Absent	Low	Bielanska et al., 2009
Colon	Colon adenocarcinoma	Positive in infiltrating inflammatory cells	Moderate (75%)	Moderate (80%)	Bielanska et al., 2009
Breast	Brast cancer	N.A.	High (30%)	N.D.	Abdul et al., 2003
			Moderate (58%)		
			Low (12%)		
	Breast adenocarcinoma	Grade I tumor	High^*^	N.D.	Brevet et al., 2009
		Grade II tumor	High^*^		
		Grade III tumor	Low^*^		
	Mammary epithelial M13SV1 cells	Inmortalized	Low ^*^	N.D.	Jang et al., 2009
	mammary epithelial m13sv1r2 cells	Weakly tumorigenic	High ^*^		
	Mammary epithelial	Highly tumorigenic	High ^*^		
	M13SV1R2-N1cells				
	Mammary duct carcinoma	Positive in infiltrating inflammatory cells	Absent	Absent/ Low (30%)	Bielanska et al., 2009
Prostate	Prostate cancer PC3, DU145, LNCaP, MDA-PCA-2B cell lines	N.A.	High (47%) Moderate (29%) Low (24%)	N.D.	Abdul and Hoosein, 2002a
	LNCaP cell lines	High K^+^ currents Weakly metastatic	High^*^	N.D.	Laniado et al., 2001
	PC3 cell lines	Low K^+^ currents strongly metastatic	Low^*^		
	AT-2 cell lines	High K^+^ currents weakly metastatic	High^*^	N.D.	Fraser et al., 2000
	Mat-LyLu cell lines	Low K^+^ currents strongly metastatic	Low^*^		
	Prostatic hyperplasia	Benign	High (89%)	N.D.	Abdul and Hoosein, 2006
	Human prostate cancer	Primary	High (52%)		
Smooth muscle	Leiomyoma	Benign	Low	Low	Bielanska et al., 2012a
	Leiomyosarcoma	Aggressive	High	Low	
Skeletal muscle	Embryonal rabdomyosarcoma	Low aggressiveness	Low	Low	Bielanska et al., 2012a
	Alveolar rabdomyosarcoma	High aggressiveness	High	High	
	Rabdomyosarcoma	N.A.	Absent	Low (30%)	Bielanska et al., 2009
Brain	Astrocytoma	Low malignancy	Low^*^	High^*^	Preußat et al., 2003
	Oligodendroglioma	High malignancy	Low^*^	Moderate^*^	
	Glioblastoma	High malignancy	Low^*^	Low^*^	
	Astrocytoma	Low malignancy	Absent/Low	Low (70%)	Bielanska et al., 2009
	Glioblastoma	High malignancy	Absent/Low	Low (40%)	
Kidney	Kidney carcinoma	N.A.	Absent	Moderate (60%)	Bielanska et al., 2009
Bladder	Bladder carcinoma	N.A.	Absent/ Low	Low (60%)	Bielanska et al., 2009
Lung	Lung adenocarcinoma	Positive in infiltrating inflammatory cells	Absent	Low (60%)	Bielanska et al., 2009
Pancreas	Pancreas adenocarcinoma	Positive in infiltrating inflammatory cells	Absent	Moderate (90%)	Bielanska et al., 2009
Ovary		Positive in infiltrating inflammatory cells	Absent	Absent	Bielanska et al., 2009
Skin	Squamous skin cell carcinoma	N.A.	Absent	High (100%)	Bielanska et al., 2009

Parenthesis indicates the percentage of expressing cells.

* Expression compared to healthy and control samples. N.D, not determined; N.A, not available.

The number of tumor cells diminishes when K^+^ channels are blocked with toxins or drugs. For example, K^+^ channel blockers exhibit anti-proliferative effects in several human cancers such as prostate tumors (Rybalchenko et al., 2001; Abdul and Hoosein, 2002a; Fraser et al., 2003), hepatocarcinoma (Zhou et al., 2003), mesothelioma (Utermark et al., 2003), colon cancer (Abdul and Hoosein, 2002b), breast carcinoma (Strobl et al., 1995; Abdul et al., 2003), glioma (Preußat et al., 2003), and melanoma (Nilius and Wohlrab, 1992; Allen et al., 1997; Artym and Petty, 2002).

### Gastrointestinal cancers

Many Kv channels, including Kv1.3, Kv1.5, Kv1.6, Kv2.1, and Kv2.2, are present in immortalized gastric epithelial cells and several gastric cancer cells (AGS, KATOIII, MKN28, MKN45, MGC803, SGC7901, SGC7901/ADR, and SGC7901/VCR). Interestingly, downregulation of Kv1.5 significantly inhibits cell proliferation and the tumorigenicity of SGC7901 cells. However, the authors conclude that Kv1.5 is necessary, but not sufficient, for gastric cancer cell proliferation (Lan et al., 2005). In addition, siRNA-mediated depletion of Kv1.5 abolished the depolarization-induced influx of Ca^2+^. Thus, Kv1.5 channels may be involved in tumor cell proliferation by controlling Ca^2+^ entry. In addition, I_Ks_ currents are related to the development of multi-drug resistance in gastric cancer cells (Wu et al., 2002). Therefore, these studies could provide a novel strategy to reverse the malignant phenotype of gastric cancer cells.

Proliferation of several human colon cancer cell lines (SW1116, LoVo, Colo320DM, and LS174t) was increased by two K^+^ channel activators, minoxidil and diazoxide. In contrast, several Kv blockers, including dequalinium and amiodarone, caused a marked growth-inhibition of human colon cancer cell lines. Glibenclamide is another Kv channel blocker that inhibits cellular proliferation (Abdul and Hoosein, 2002a,b). Proliferation of the colorectal carcinoma cell line DLD-1 is drastically reduced in the presence of 4-AP. However, inhibition of Ca^2+^-sensitive K^+^ channels and ATP-sensitive K^+^ channels did not have an effect on cell proliferation. Interestingly, K^+^ channel inhibitors blocked [Ca^2+^]_i_ influx, suggesting that K^+^ channel activity may control the proliferation of colon cancer cells by modulating Ca^2+^ entry (Yao and Kwan, 1999). Although colon biopsies exhibited an increase in Kv1.3 and Kv1.5 expression, this phenomenon may be an artifact of the massive presence of inflammatory cells, which express high levels of both channels (Bielanska et al., 2009).

### Breast cancer

Kv1.3 expression has been examined by immunohistochemistry in healthy human breast samples and their matched cancer tissue counterparts. While Kv immunostaining is not observed in normal human breast tissues, most cancer specimens show moderate staining in the epithelial compartment. In addition, the K^+^ channel activator minoxidil stimulates the growth of MCF-7 human breast cancer cells. On the contrary, K^+^ channel blockers such as dequalinium and amiodarone have marked inhibitory effects on MCF-7 cell proliferation (Abdul et al., 2003). Other K^+^ channel-blockers also inhibit breast cancer growth (Strobl et al., 1995) and potentiate the growth-inhibitory effects of tamoxifen on human breast (MCF-7, MDA-MB-231), prostate (PC3, MDA-PCA-2B), and colon (Colo320DM, SW1116) cancer cell lines (Abdul et al., 2003). However, the expression of Kv1.3 in breast cancer is not well defined. Kv1.3 expression is increased in breast cancer biopsies in comparison with healthy breast tissues (Abdul et al., 2003). However, Brevet et al. argues that less Kv1.3 expression is found in cancerous samples, and claims that Kv1.3 gene promoter methylation is increased. Because Kv1.3 expression correlates with both poorly differentiated tumors and a younger age of patients with tumors, the authors suggest that a loss of Kv1.3 may be a marker for poor prognosis of breast tumors (Brevet et al., 2009). Immortalized human mammary epithelial cells with different tumorigenic properties demonstrated that the expression of Kv1.3 varies depending on the tumorigenicity and stage of the breast cancer (Jang et al., 2009). In addition, we have recently found that Kv1.3 and Kv1.5 expression increases concomitantly with an elevation of infiltrating inflammatory cells surrounding the tumor nodule in breast carcinoma samples (Bielanska et al., 2009). This finding could shed light on the debate, however, more studies must be undertaken to elucidate these mechanisms.

### Prostate cancer

Expression of Kv1.3 and Kv1.5 channels has also been extensively studied in prostate cancer cells. Four different human prostate cancer (Pca) cell lines, two of which were androgen-unresponsive (PC3, DU145) and two of which were androgen-responsive (MDA-PCA-2B, LNCaP), were examined by immunohistochemistry to determine expression levels of Kv1.3. Strong immunostaining for Kv1.3 was detected in normal prostate samples, whereas most of the Pca specimens showed strong and moderate Kv1.3 staining (Abdul and Hoosein, 2002a). In addition, different K^+^ channel activators, such asminoxidil, 1-ethyl-2-benzimidazolinone, and diazoxide, had significant growth-stimulatory effects on PC3 cells. In contrast, K^+^ channel-blockers such as dequalinium, amiodarone, and glibenclamide, caused a dose-dependent growth inhibition of both androgen-unresponsive and androgen-responsive Pca cell lines. Furthermore, channel blockers triggered morphological feature changes such as nuclear shrinkage and fragmentation, suggesting an activation of apoptotic signaling mechanisms (Abdul and Hoosein, 2002a). Although the highly metastatic PC3 cell line expressed Kv1.3 (Laniado et al., 2001), its expression was inversely related to metastasis in prostate cancer (Abdul and Hoosein, 2002a). In another report, Kv density inversely correlated with the metastasis of human prostate cancer cell lines (Laniado et al., 2001). Thus, lower Kv-staining in clinical Pca specimens compared to Kv-staining levels in normal prostate cells may correlate with an increased probability of metastatic disease (Abdul and Hoosein, 2002a). Voltage-gated K^+^ currents have been characterized by electrophysiology in rat (Mat-LyLu and AT-2) and human (PC3 and LNCaP) PCa cell lines (Skryma et al., 1997; Rane, 2000; Laniado et al., 2001; Rybalchenko et al., 2001). Both the strongly metastatic MAT-LyLu and the weakly metastatic AT-2 cell lines expressed Kv1.3 currents. Interestingly, Kv1.3 currents had different biophysical properties in the two rat prostate cancer cell lines, which displayed markedly different metastatic abilities. Thus, MAT-LyLu cells displayed significantly smaller maximal K^+^ current densities and an increased negative resting potential when compared to AT-2 cells. Taken together, these data suggest that K^+^ currents in the MAT-LyLu cells may be less active than those in the AT-2 cells (Fraser et al., 2000). Therefore, human prostate cancer cells with different metastatic ability displayed a differential modulatory action of K^+^ channels. This finding, together with the exclusive expression of voltage-gated Na^+^ channels in MAT-LyLu cells (Grimes et al., 1995), suggests a role for voltage-dependent ion channels in metastatic cell behavior (Laniado et al., 1997; Fraser et al., 2003). High epithelial Kv1.3 expression has also been observed in all normal prostate and benign prostatic hyperplasias (BPH), whereas only half of primary human prostate cancer (Pca) samples express Kv1.3. Furthermore, reduced Kv1.3 protein levels in Pca correlated with high tumor grade and a poor prognosis. Because there was a significant inverse correlation between Kv1.3 levels and prostate tumor stage, Kv1.3 expression may be a useful diagnostic or prognostic marker for prostate cancer (Abdul and Hoosein, 2006).

### Muscle sarcomas

Kv channels are crucial for the modulation of arterial tone and the control of vascular smooth muscle cell proliferation (Michelakis et al., 1997) and migration (Cidad et al., 2010; Cheong et al., 2011). Although myofibers are terminally differentiated, some myoblasts may proliferate by re-entering the cell cycle. Margatoxin, a specific blocker of Kv1.3, reduces proliferation and migration of mouse and human vascular smooth muscle cells. However, margatoxin does not fully abrogate migration, suggesting that a Kv1.3-independent component is involved in this process (Cheong et al., 2011). During vascular smooth muscle proliferation, Kv1.3 expression increases while Kv1.5 expression decreases (Cidad et al., 2010). Thus, Kv1.3 expression is altered during myoblast proliferation and differentiation, although it does not play a substantial role in either process (Villalonga et al., 2008). Conversely, Kv1.5 channel expression seems to contribute to vascular smooth muscle tone (Yuan et al., 1998).

In a recent study, we analyzed Kv1.3 and Kv1.5 expression in human samples of smooth muscle tumors [such as leiomyoma (LM) and leiomyosarcoma (LMS)] and compared the tumor samples to their healthy specimen counterparts. LM and LMS are a benign uterus tumor and an aggressive retroperitoneal neoplasm, respectively. Kv1.3 is poorly expressed in healthy muscle and in indolent LM samples but was significantly induced in malignant LMS. Similar to Kv1.3, Kv1.5 is almost absent in healthy biopsies, but Kv1.5 staining was heterogeneous and faint in LM samples. In contrast, Kv1.5 displayed a poor and homogeneous expression in aggressive LMS samples. Interestingly, a clear positive correlation between the expression of Kv1.3 and Kv1.5 and the aggressiveness of the smooth muscle neoplasm was noted. These results suggested that Kv1.3 and Kv1.5 could be used as potential molecular targets for the treatment of aggressive smooth muscle sarcomas (Bielanska et al., 2012a,b).

Kv1.3 and Kv1.5 undergo alterations in different types of human skeletal muscle sarcomas (Bielanska et al., 2009, 2012b). Kv1.3 is absent in the less aggressive embryonal rhabdomyosarcoma (ERMS), whereas its expression in the aggressive alveolar rhabdomyosarcoma (ARMS) is notable and equivalent to that found in fetal muscle. Kv1.5, which weakly stains healthy adult skeletal muscle, strongly stains fetal tissues in a manner similar to that of Kv1.3. In addition, ERMS specimens show heterogeneous expression of Kv1.5, which could indicate different stages of proliferation and/or differentiation of individual cells. In contrast, ARMS samples express homogeneous and highly expressed levels of Kv1.5 (Bielanska et al., 2012b). Therefore, the expression of both Kv1.3 and Kv1.5 channels increased significantly with respect to the tumor aggressiveness grade in ERMS and ARMS.

### Gliomas

In addition to contributing to the proliferation of normal glia, Kv1.3 is detected in human gliomas, which are brain tumors arising from glial cells. Gliomas emerge from both astrocytic and oligodendrocytic lineages and consist of low and high malignancy grades, respectively. Preußat and coworkers have reported that some Kv1.3 and Kv1.5 differential expression occurs with respect to the malignancy grade of the tumor. For example, Kv1.5 expression is elevated in astrocytomas, moderate in oligodendrogliomas, and low in glioblastomas. Although the expression of Kv1.5 inversely correlates with glioma malignancy, no such correlation is evident for Kv1.3. These data suggest that reduced levels of Kv1.5 protein in biopsies when compared to the levels found in adjacent healthy tissues may be a good candidate biomarker for both glioma detection and outcome prediction (Preußat et al., 2003). Other studies have revealed abundant Kv1.3 and Kv1.5 expression in brain tumors and suggested that while Kv1.3 expression is notable in astrocytomas, Kv1.5 expression is elevated in glioblastomas (Bielanska et al., 2009). Although it is not known whether Kv1.3 and Kv1.5 expression is increased in gliomas vs. healthy cells, Kv1.5 expression also occurred more in diffuse astrocytoma than in high grade ones. Moreover, glioblastoma patients with higher Kv1.5 expression had slightly better survival (Arvind et al., 2012).

### Other solid cancers

Recently, we have performed an extensive analysis of Kv1.3 and Kv1.5 protein expression in a wide variety of human tumors. Our results indicated that most cancers experienced an alteration of Kv gene expression. We found that Kv1.3 is present in healthy stomach, kidney, skeletal muscle, and lymph node, whereas expression of Kv1.3 was low in the breast, ovary, pancreas, bladder, lung, colon, and brain. Taken together, these data demonstrated that Kv1.3 is more ubiquitously expressed than was suggested by previous studies. In addition, most tumors showed no major differences in Kv1.3 expression when compared to healthy tissues. However, Kv1.3 expression was downregulated in kidney, bladder, and lung carcinomas (Bielanska et al., 2009). On the other hand, Kv1.5 expression was evident in most of the analyzed tissues but not in the breast. The abundance of Kv1.5 was low in the ovary, urinary bladder, and lung. Interestingly, unlike Kv1.3, Kv1.5 expression was increased in most human tumors. For example, stomach, pancreatic, and bladder tumors expressed more Kv1.5 than healthy specimens, however, Kv1.5 expression was decreased in renal adenocarcinoma when compared to healthy tissues. Because this channel is involved in K^+^ transport and cell volume regulation (Felipe et al., 1993), a decrease in Kv1.5 expression would likely be accompanied by a loss of renal function. Finally, Kv1.5 expression was unaffected in ovary and lung tumors (Bielanska et al., 2009).

The immunohistochemical analysis of Kv1.3 and Kv1.5 channels in all of these cancers demonstrated that in most cases, stronger Kv1.3 and Kv1.5 expression is mainly confined to the inflammatory cells surrounding the tumors (Bielanska et al., 2009).

It is tempting to speculate that because these human specimens are usually from patients who have already been diagnosed with some type of cancer, most of them show histological signs of reactivity and should be interpreted with caution. This was the case for stomach, pancreatic, and breast cancers (Bielanska et al., 2009) and it may explain differences between our studies and those performed by Hoosein and coworkers in breast cancer (Abdul et al., 2003). Contrary to this hypothesis, other cancers such as those of the bladder, skin, ovary, and lymph node, exhibited Kv1.5 induction in the tumorigenic cells (Bielanska et al., 2009).

## Kv1.3 and Kv1.5 in blood cancers

### Lymphomas

Because Kv channels control neoplastic processes in leukocytes such as cell activation, proliferation, migration and apoptosis (DeCoursey et al., 1984; Gollapudi et al., 1988; Khanna et al., 1999; Wickenden, 2002; Vicente et al., 2003, 2005; Cahalan and Chandy, 2009; Wulff et al., 2009), these proteins are thought to be involved in the mechanisms underlying lymphoma invasiveness (Cruse et al., 2006; Bielanska et al., 2009; Felipe et al., 2012) and malignancy.

**Figure 1:**
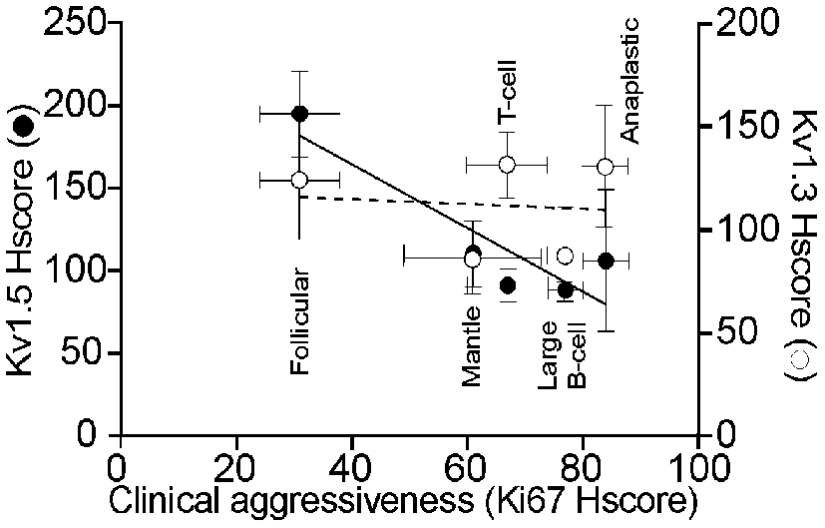
**Expression of Kv1.3 and Kv1.5 in human non-Hodgkin lymphomas.** A Histoscore (Hscore) was calculated to establish a phenotypical correlation of clinical agressiveness based in the expression of Ki67 (Castellvi et al., 2009). (◦) Kv1.3 Hscore in non-Hodgkin's lymphomas. (•) Kv1.5 Hscore. Results are mean ± SEM of Kv1.3 and Kv1.5 Hscore plotted against Ki67 Hscore as a marker of the clinical aggressiveness of the lymphoma. While a Pearson's correlation coefficient of *r* = 0.106 with a *P* < 0.866 indicated a complete absence of correlation of Kv1.3, a Pearson's correlation coefficient of *r* = 0.895 with a *P* < 0.040 indicated an inverse correlation between Kv1.5 abundance and the aggressiveness of tumor.

In a preliminary study, we have shown that Kv1.3 and Kv1.5 are differentially altered in human non-Hodgkin's lymphomas (Bielanska et al., 2009), however, a more complete study was required to confirm these initial findings. Recently, we have examined the expression of Kv1.3 and Kv1.5 in a panel of human non-Hodgkin lymphomas. To our knowledge this was the first study to examine Kv1.3 and Kv 1.5 expression in diffuse large B-cell, follicular B-cell, mantle, anaplastic and T-cell lymphomas in comparison with control lymph nodes. Furthermore, because these human cancers exhibited different grades of malignancy, we determined whether there was a correlative relationship between Kv1.3 and Kv1.5 expression and the clinical aggressiveness of these human lymphomas (Figure 1). Kv channels have previously been proposed as tumorigenic markers and therapeutic targets (Conti, 2004; Kunzelmann, 2005; Pardo et al., 2005; Felipe et al., 2006; Stuhmer et al., 2006), although in most cases there was no clear correlation between channel expression and tumorigenicity (Preußat et al., 2003). In these studies, we found that control lymph nodes expressed high levels of heterogeneous Kv1.3, which could indicate a certain mechanism of action, while Kv1.5 abundance was low and homogeneous. Interestingly, Kv1.3 and Kv1.5 were differentially altered in non-Hodgkin's human lymphomas. For example, indolent follicular lymphomas expressed noticeable levels of Kv1.5, while aggressive diffuse large B cell lymphomas showed low Kv1.5 expression. Thus, Kv1.5 expression is inversely correlated with clinical aggressiveness in non-Hodgkin's lymphomas. Preuβ at and coworkers found a similar inverse correlation between the level of Kv1.5 immunostaining and tumor grade in gliomas (Preußat et al., 2003). Although further studies with a larger number of subjects for each tumor type must be performed, the level of Kv1.5 protein may be useful in the diagnosis or prognosis of some lymphomas (Table 2).

**Table 2:** **Expression of Kv1.3 and Kv1.5 in blood cancers**.

**Tissue**	**Kv1.3**	**Kv1.5**	**Tumors and Cell lines**	**Features**	**References**
Lymph node	Low	High	Follicular B-cell lymphoma	Low aggressiveness non-Hodgkin's lymphoma	Vallejo-Gracia et al., 2013
Lymph node	Low	Low	Mantle lymphoma	High aggressiveness non-Hodgkin's lymphoma	Vallejo-Gracia et al., 2013
Lymph node	Low	Moderate	T-cell lymphoma	High aggressiveness non-Hodgkin's lymphoma	Vallejo-Gracia et al., 2013
Lymph node	Moderate	Low	Diffuse large B-cell lymphoma	High aggressiveness non-Hodgkin's lymphoma	Vallejo-Gracia et al., 2013
Lymph node	Low	Moderate	Anaplastic lymphoma	High aggressiveness non-Hodgkin's lymphoma	Vallejo-Gracia et al., 2013
Lymph node	High	N.D.	Chronic lymphocytic leukenia		Leanza et al., 2013

N.D, not determined.

In contrast, the expression of Kv1.3, did not correlate with either the state of de-differentiation or the nature of the lymphomas, although its expression was decreased in most cancers (Bielanska et al., 2009) (Figure 1). Previous studies have also demonstrated that Kv1.3 expression showed no apparent connection with the tumorigenic state when considering the prognosis of the tumor (Arcangeli et al., 2009; Bielanska et al., 2009). Kv1.3 expression showed no apparent correlation with malignancy or clinical aggressiveness, similar to the findings in gliomas (Preußat et al., 2003). Taken together, these data suggest that Kv1.3 may act as a tumor suppressor. Hypoxia, which occurs commonly in solid tumors and is associated with malignant progression (Vaupel et al., 2004), decreased Kv1.3 protein levels and activity in human T lymphocytes (Conforti et al., 2003). Moreover, suppression of Kv1.3 prevents apoptosis, which would favor tumor development (Bonnet et al., 2007).

### Leukemias

Distinct K^+^ channel blockers have anti-proliferative effects on human myeloblastic leukemia cells (Wang et al., 1997; Xu et al., 1999). Moreover, membrane-permeable K^+^ channel inhibitors, such as Psora-4, PAP-1 and clofazimine, induce apoptosis of chronic lymphocytic leukemia cells. In contrast, these cells are resistant to the membrane-impermeable inhibitor ShK, which clearly suggests that the plasma membrane-located Kv1.3 is not responsible for the observed apoptotic response. In fact, pathologic B cells showed higher Kv1.3 protein expression and were sensitive to treatment, whereas healthy cells express less Kv1.3 and were resistant to the drugs (Leanza et al., 2013). Clofazimine treatment also significantly reduced tumor size in an orthotopic melanoma mouse model (Leanza et al., 2012). Therefore, clofazimine might be a promising new therapeutic tool to treat chronic lymphocytic leukemia patients (Leanza et al., 2013).

Because Kv1.3 expression was decreased in most cancers, some authors have suggested that this channel may act as a tumor suppressor. In this context, hypoxia decreased Kv1.3 protein levels and inhibited proliferation of T-lymphocytes (Conforti et al., 2003; Chandy et al., 2004; Vaupel et al., 2004). Surprisingly, the Kv1.3 channel is also thought to play an important role in apoptosis in T-cells (Arcangeli et al., 2009) because elevated Kv1.3 facilitates an apoptotic response (Bock et al., 2002; Szabo et al., 2008). Thus, it is thought that Kv1.3 promotes proliferation in oligodendrocytes (Vautier et al., 2004) but also controls leukocyte activation and is crucial for the induction of apoptosis in lymphocytes (Storey et al., 2003; Szabo et al., 2004, 2008, 2010; Gulbins et al., 2010). These interesting findings seem rather contradictory with respect to the cellular function of Kv1.3. However, it has been suggested that the environmental conditions in which channel activation takes place and the magnitude of the activated conductance could determine whether the channel supports proliferation or apoptosis (Kunzelmann, 2005). In this context, it is tempting to speculate that suppression of Kv1.3 activity would slow apoptosis and favor tumor development. Moreover, increasing the expression of Kv1.5 with dichloroacetate triggers apoptosis in lung, breast, glioblastoma and endometrial cancer cell lines (Bonnet et al., 2007; Wong et al., 2008). Furthermore, the K^+^ channel blocker clofilium induces apoptosis in human promyelocytic leukemia (HL-60) cells (Choi et al., 1999). However, these hypotheses are supported by little evidence, and further research is required to confirm these conclusions.

## Conclusion

Several K^+^ channels are essential for cell proliferation and appear to play a role in the development of cancer. In this context, further investigation is needed to fully understand the role of membrane ion channels in normal and neoplastic cell proliferation. A large body of data indicates that tumor cells differentially altered the expression of voltage dependent K^+^ channels. Furthermore, Kv1.5 and to some extent Kv1.3, are aberrantly expressed in many human cancers. We can conclude that the abundance of Kv1.5 expression mostly increases in tumor cells, whereas Kv1.3 expression is generally downregulated. Interestingly, both Kv1.3 and Kv1.5 have displayed an apparent connection between their expression and the tumorigenic state of different cancer cells that may be attributable to remodeling mechanisms. To date we know that there is a clear positive correlation between the expression of both Kv1.3 and Kv1.5 channels and the clinical aggressiveness of smooth muscle neoplasms. In contrast, an inverse correlation between the levels of Kv1.3 and prostate cancer tumor stage/metastatic capacity and an inverse correlation between Kv1.5 expression and glioma tumor grade have been described. In addition, Kv1.5 expression exhibits a significant correlation with the degree of malignancy of rhabdomyosarcomas, renal tumors and lymphomas. These findings suggest that Kv1.3 and Kv1.5 channels could be used not only as tumor biomarkers but also as prognostic and diagnostic indicators.

### Conflict of interest statement

The authors declare that the research was conducted in the absence of any commercial or financial relationships that could be construed as a potential conflict of interest.
